# Spatial disparity in the distribution of superfund sites in South Carolina: an ecological study

**DOI:** 10.1186/1476-069X-12-96

**Published:** 2013-11-06

**Authors:** Kristen Burwell-Naney, Hongmei Zhang, Ashok Samantapudi, Chengsheng Jiang, Laura Dalemarre, LaShanta Rice, Edith Williams, Sacoby Wilson

**Affiliations:** 1 Maryland Institute for Applied Environmental Health (MIAEH), School of Public Health, University of Maryland, College Park, MD, USA; 2 Community Engagement, Environmental Justice, and Health (CEEJH), University of Maryland, College Park, MD, USA; 3 Department of Epidemiology and Biostatistics, Arnold School of Public Health, University of South Carolina, Columbia, SC, USA; 4 Department of Health Promotion, Education, and Behavior, Arnold School of Public Health, University of South Carolina, Columbia, SC, USA; 5 Institute for Partnerships to Eliminate Health Disparities, University of South Carolina, Columbia, SC, USA

## Abstract

**Background:**

According to the US Environmental Protection Agency (EPA), Superfund is a federal government program implemented to clean up uncontrolled hazardous waste sites. Twenty-six sites in South Carolina (SC) have been included on the National Priorities List (NPL), which has serious human health and environmental implications. The purpose of this study was to assess spatial disparities in the distribution of Superfund sites in SC.

**Methods:**

The 2000 US census tract and block level data were used to generate population characteristics, which included race/ethnicity, socioeconomic status (SES), education, home ownership, and home built before 1950. Geographic Information Systems (GIS) were used to map Superfund facilities and develop choropleth maps based on the aforementioned sociodemographic variables. Spatial methods, including mean and median distance analysis, buffer analysis, and spatial approximation were employed to characterize burden disparities. Regression analysis was performed to assess the relationship between the number of Superfund facilities and population characteristics.

**Results:**

Spatial coincidence results showed that of the 29.5% of Blacks living in SC, 55.9% live in Superfund host census tracts. Among all populations in SC living below poverty (14.2%), 57.2% were located in Superfund host census tracts. Buffer analyses results (0.5mi, 1.0mi, 5.0mi, 0.5km, 1.0km, and 5.0km) showed a higher percentage of Whites compared to Blacks hosting a Superfund facility. Conversely, a slightly higher percentage of Blacks hosted (30.2%) a Superfund facility than those not hosting (28.8%) while their White counterparts had more equivalent values (66.7% and 67.8%, respectively). Regression analyses in the reduced model (Adj. R^2^ = 0.038) only explained a small percentage of the variance. In addition, the mean distance for percent of Blacks in the 90th percentile for Superfund facilities was 0.48mi.

**Conclusion:**

Burden disparities exist in the distribution of Superfund facilities in SC at the block and census tract levels across varying levels of demographic composition for race/ethnicity and SES.

## Background

The Superfund program was established by the United States Environmental Protection Agency (USEPA) to address abandoned hazardous waste sites [1]. These abandoned sites are thought to pose a significant threat to human health and the environment, and as a result, may qualify for placement on the USEPA’s Superfund list to receive federal cleanup funds [2]. Currently, there are more than 1,200 sites on the USEPA’s National Priorities List (NPL), which is comprised of the country’s most serious hazardous waste sites that are eligible for cleanup under the Superfund program [1]*.* As part of the federal mandate, the USEPA was tasked with locating and ranking the most severe Superfund sites for remedial action [1]. In order for a Superfund site to be placed on the NPL, the following procedures must be implemented: 1) an alleged hazardous waste site must be proposed to the USEPA, 2) public comments must be accepted for the site, and 3) the USEPA must respond to the comments and places the sites on the NPL that meet certain inclusion criteria [1].

### Environmental justice

The geographic distribution of Superfund sites has always been a controversial issue because research has shown that hazardous waste sites are differentially located in predominately Non-White and low-income communities. An environmental justice (EJ) analysis conducted by Maranville et al., examined whether the presence of a Superfund site affected surrounding communities in the state of Illinois in order to inform future siting decisions and improve current sites [3]. Geographic Information Systems (GIS) was used to create one, two, and five mile buffer zones around Superfund sites to capture the sociodemographic composition of host communities [3]. The study found that percent Non-White was significantly higher than the percent of White populations within a one mile radius surrounding the Superfund sites [3]. Furthermore, over 50% (24/43) of the sites included in the analysis had a higher percentage of Non-White populations residing near the environmental hazards [3]. The aforementioned results suggest that race/ethnicity may be the principal driver of environmental inequity.

The objectivity of the Superfund program has been questioned due to the disproportionate number of Non-White and low-income populations that may not be benefiting from cleanup efforts [2]. While there are certain criteria that determine whether a site is placed on the NPL, such as the severity of the hazard, or if the site presents less of a hazard thus making the cleanup process less arduous; there are additional racial and socioeconomic determinants that may influence the fate of a site [3]. A 2007 study by O’Neil [2] examined the relationship between environmental remediation and EJ by evaluating the impact of Executive Order 12898 [4,5] on the Superfund listing and cleanup process.

O’Neil found that a one percent increase in Non-White populations was associated with a 0.2% decrease in the probability of a Superfund listing [2]. The results of the study suggest that for sites discovered after the 1994 Executive Order 12898, there was a lower chance of a Superfund listing for poor communities and disadvantaged communities of color [2]. Despite the EJ Executive Order, equity in the Superfund listing process worsened after 1994 [2]. In addition, it appears that the USEPA has failed to properly implement Executive Order 12898 in regards to the Superfund program [2] particularly in EJ communities known to have a high concentration of hazardous waste sites.

### Environmental effects

Some of the common contaminants found at Superfund sites are asbestos, dioxin, and mercury, all of which may pose a significant threat to ecological health. Asbestos is a naturally occurring fibrous silicate mineral that has been mined for its invaluable properties and used in many commercial products that include insulation, brake linings, and roofing shingles [6]. Moreover, asbestos may enter the air and water from the weathering of natural deposits and the decomposition of manufactured products (e.g., brake pads) [6]. Small fibers may remain suspended in the air for an extended period of time before settling which may increase the duration of exposure [7].

Dioxin refers to a group of toxic chemical compounds that share certain chemical structures and biological characteristics [8]. Dioxins may be very toxic to certain animals as well as humans, particularly during their early stages of development when the body is less capable of metabolizing the aforementioned compound. While, mercury is another naturally occurring chemical in the environment, additional sources include coal and oil combustion as well as emissions from incineration and landfills. These emissions may contaminate soil and water, which can lead to deleterious effects on various animal species such as loons, eagles, otters, mink, and kingfishers [9].

### Health effects

Despite the limitations in exposure science to link Superfund site contaminants with long-term health effects, there have been studies to show the detriment of volatile organic compounds (VOCs) that were released in drinking water among Superfund host communities [10]. The adverse health effects attributable to VOC exposure included the following: (1) birth defects, (2) diabetes, (3) urinary tract disorders, (4) eczema and skin conditions, (5) anemia, (6) speech and hearing difficulties in young children, and (7) stroke [10]. Moreover, a study that evaluated local health problems and exposure to heavy metals at the Tar Creek Superfund site in Ottawa County, Oklahoma found an increase in mortality incidence for heart disease among adults as well as increased blood lead levels (>10 μg/dl) in over 50% of the children which exceeded normal intake standards [11]. Another study by Williamson et al. found that people who live near multiple Superfund sites were more likely to have immunoglobulin test results that are lower or higher than the reference range when compared to populations further away from these sites or other environmental hazards [12]. The major implications of having abnormal immunoglobulin levels is that it decreases immune function, which then impairs the body’s ability to protect against disease [12]. As a result, populations living in close proximity to Superfund sites may be more susceptible to chronic and infectious diseases as well as those related to chemical exposures.

### Property values

The proximity of Superfund sites to neighboring communities, whether commercial or residential may have a drastic effect on property values [13]. Properties located close to these sites may depreciate due to unwanted land uses [14]. Unfortunately, there is little that a homeowner can do to reduce their exposure to nearby waste sites since it is the responsibility of the company to ensure that harmful chemicals are not released into the community. If these hazardous chemicals were dispersed into the environment, they could pose a serious health threat to the community and surrounding property. Specifically, the area may be deemed unlivable due to irreversible contamination of soil or pollution of surface waters and drinking water resources [13].

While research has shown that hazardous waste sites are located in predominately Non-White and low-income communities, there is a paucity of research describing the profile of populations hosting those sites, particularly in the state of South Carolina (SC). The purpose of this study was to evaluate the spatial distribution of Superfund sites in SC across areas with varying racial/ethnic and socioeconomic composition.

## Methods

### Superfund sites in South Carolina

Superfund data was obtained from the USEPA’s Comprehensive Environmental Response, Compensation, and Liability Information System (CERCLIS) public access database, which contains “non-enforcement confidential” information on hazardous waste sites, potentially hazardous waste sites, and remedial activities as well as those noted on the NPL [15]. As of December 2011, SC has 274 active Superfund sites [15] that were used in the analysis and mapped based on the addresses provided in the USEPA’s CERCLIS Public Access Database.

### Sociodemographic status

This study used 2000 US Census Bureau sociodemographic data derived from summary files 1 and 3. While demographic information is available at various geographic scales (ZIP code tabulation areas (ZCTAs), tracts, block groups, and blocks); we utilized census data at the tract and block level to enumerate the following population characteristics: race/ethnicity (% White, % Black, % Non-White) and socioeconomic status (SES). SES variables considered in our study included poverty (% of population below poverty line), education (% of population with less than a high school education), unemployment (% of population who are unemployed), homeowners (% of population who own a home), and home built before 1950 (% of population who own a home built before 1950). Other SES-related variables included % Black below poverty level, and % Black with less than a high school education. The same summary statistics (% below poverty and % having less than a high school education) were obtained for White populations as well. The variables related to income used in this study were per-capita income and median household income.

### Statistical analyses

We used SAS version 9.2 (SAS Institute Cary, NC) to perform statistical analyses to assess disparities in the prevalence of these hazardous sites across areas with varying racial/ethnic and SES composition. Buffer analysis techniques were used to address burden disparities in the distribution of Superfund sites across SC at the census block and tract level. Buffer distances of 0.5, 1.0 and 5.0 miles (mi) as well as 0.5, 1.0, and 5.0 kilometers (km) were created. The population size, percent Black, and percent Non-White were calculated for each buffer distance. The mean and median distance of census tracts to the nearest Superfund site was calculated for each sociodemographic factor at the 10^th^, 25^th^, 50^th^, 75^th^ and 90^th^ percentiles. Summary statistics were calculated for all variables and the graphical tool gnuplot version 4.6 was used to visualize the association of the percentage of all variables with distance to the nearest Superfund site.

Chi-square tests were used to evaluate the difference in the proportion of populations who host a Superfund site and compared to those not hosting a Superfund site for all sociodemographic factors. Specifically, chi-square tests were conducted for each buffer distance for all sociodemographic factors to determine the difference in the proportion of populations who host a Superfund site compared to those that do not host Superfund sites. Linear regression models were applied to describe the relationship between the distance to the nearest Superfund site (dependent variable) and the sociodemographic factor (independent variables). Selection of all significant variables was conducted using the backward selection model. For variables related to income (per capita income, median household income), we performed t-tests to determine whether they were significantly different from populations hosting a Superfund site and those not hosting a Superfund site. In all the analyses, the overall significance level for each type of test was set at 0.05.

## Results

The descriptive statistics for all sociodemographic factors across census tracts are included in Table 1 where the results summarized as means calculated over all census tracts in the state. On average, 32.4% of the population in a census tract was Black while 64.4% were White. Approximately 15.8% of the population was living below poverty line, which is higher than the national average of 11.3% in 2000. Those with less than a high school education comprised 25.1% of the population and the unemployment rate was 3.9%. When considering housing characteristics, 69.6% of the population owned their own home and 13.6% built their home before 1950. In addition, 6.1% of the White population lived below poverty line while 5.1% of Blacks live below poverty line. These variables were calculated with respect to the entire state, which has a much larger White population than Black. Therefore, percent poverty was automatically higher among Whites when compared with their Black counterparts.

**Table 1:** Summary statistics for sociodemographic factors in the State of South Carolina (US Census 2000)

**Variable**	**Minimum**	**Lower Quartile**	**Mean**	**Upper quartile**	**Maximum**	**Median**
% Black	0.04	11.16	32.42	49.69	98.83	26.05
% Non-White	0.74	14.50	35.59	52.48	99.57	30.23
% White	0.43	47.51	64.41	85.50	99.26	69.76
% Poverty	0.00	8.20	15.78	20.60	100.00	13.60
% Homeowner	0.00	61.40	69.63	84.40	100.00	75.50
% < HS Education	0.00	14.60	25.13	33.90	63.10	26.90
% Homes Built Before 1950	0.00	4.20	13.61	18.70	100.00	9.50
% Unemployed	0.00	2.25	3.91	4.70	53.93	3.30
% Poverty White	0.00	3.26	6.05	7.67	60.91	5.26
% Poverty Black	0.00	0.16	5.11	6.72	58.51	1.38
% White with < HS Education	0.00	1.47	4.53	6.52	23.94	3.56
% Black with < HS Education	0.00	0.21	6.17	8.53	55.37	1.80

Note: The summaries of quartiles are with respect to 25%, 50%, and 75% of census tracts. For example, for % Poverty White, the lower quartile of 3.26 indicates that 25% of the tracts have at most 3.26% of Whites in poverty.

**Figure 1:**
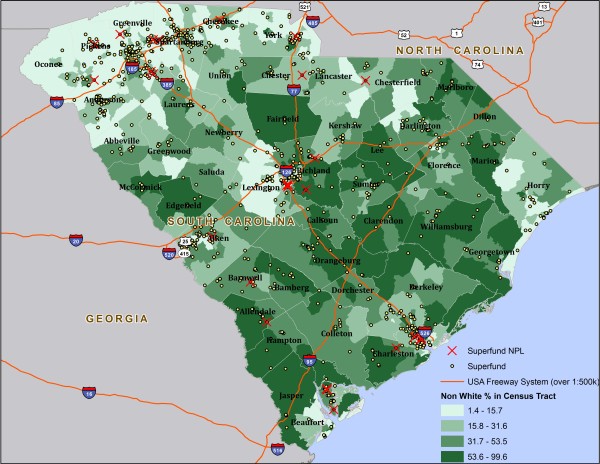
Map of Superfund Sites and NPL Sites in South Carolina by Percent Non-White (US Census 2000).

**Figure 2:**
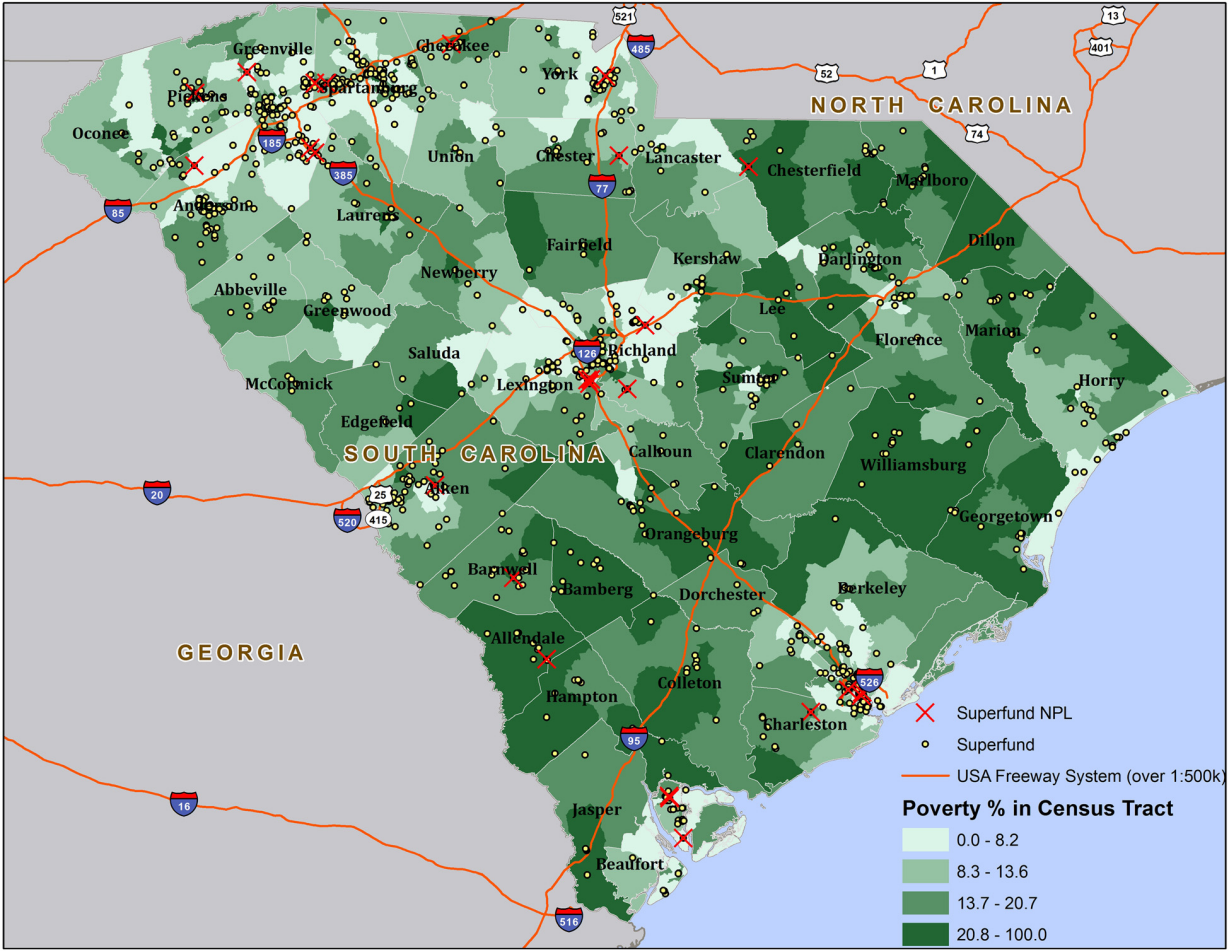
Map of Superfund Sites and NPL Sites in South Carolina by Percent Poverty (US Census 2000).

Choropleth maps were then drawn to describe the distribution patterns of Superfund sites across demographic variables of interest. Figure 1 is a choropleth map constructed in ArcGIS 10.0 to illustrate the spatial distribution of Superfund sites in relation to percent Non-White within the state. The values of percent Non-White were divided into four quartiles and were represented by different colors in the graph. There were large clusters of Superfund sites located in densely populated areas in the Northwest, west, central, and coastal regions of the state. These clusters appear to be concentrated in high population density counties such as Greenville, Spartanburg, Anderson, Richland, Charleston, Pickens, and Aiken. While the Northeast region has multiple Superfund clusters, this part of the state has the highest percentage of Whites and lowest number of Superfund sites. The choropleth map for the distribution pattern of Superfund sites in relation to percent poverty across the state highlighted similar points (Figure 2).

Large clusters of Superfund sites were observed and located in population areas of the state such as Greenville, Spartanburg, Anderson, Richland, Charleston, Pickens, and Aiken. These counties are mostly comprised of more affluent areas and have fewer Superfund sites compared to the high poverty areas in the state. In addition, we observed that as percent poverty increased, the number of Superfund sites increased.

The mean and median distances between all census tracts for SC and the nearest Superfund site were 0.46 and 0.00mi, respectively. The mean distance between the nearest Superfund site and census tracts where the Non-White population was greater than 50% was 0.48mi and the mean distance between the nearest Superfund site and census tracts where Whites accounted for more than 50% of the population was 0.45mi. Among census tracts where population sizes of Blacks were in the first quartile (25^th^ percentile), the mean distance was 0.49mi from the nearest Superfund site to these census tracts. The distance decreased to 0.45mi among census tracts in the third quartile (75^th^ percentile). The median distance for 25^th^ and 75^th^ percentiles was 0.06 and 0.00mi, respectively, which implies that most Blacks were within Superfund host areas.

**Figure 3:**
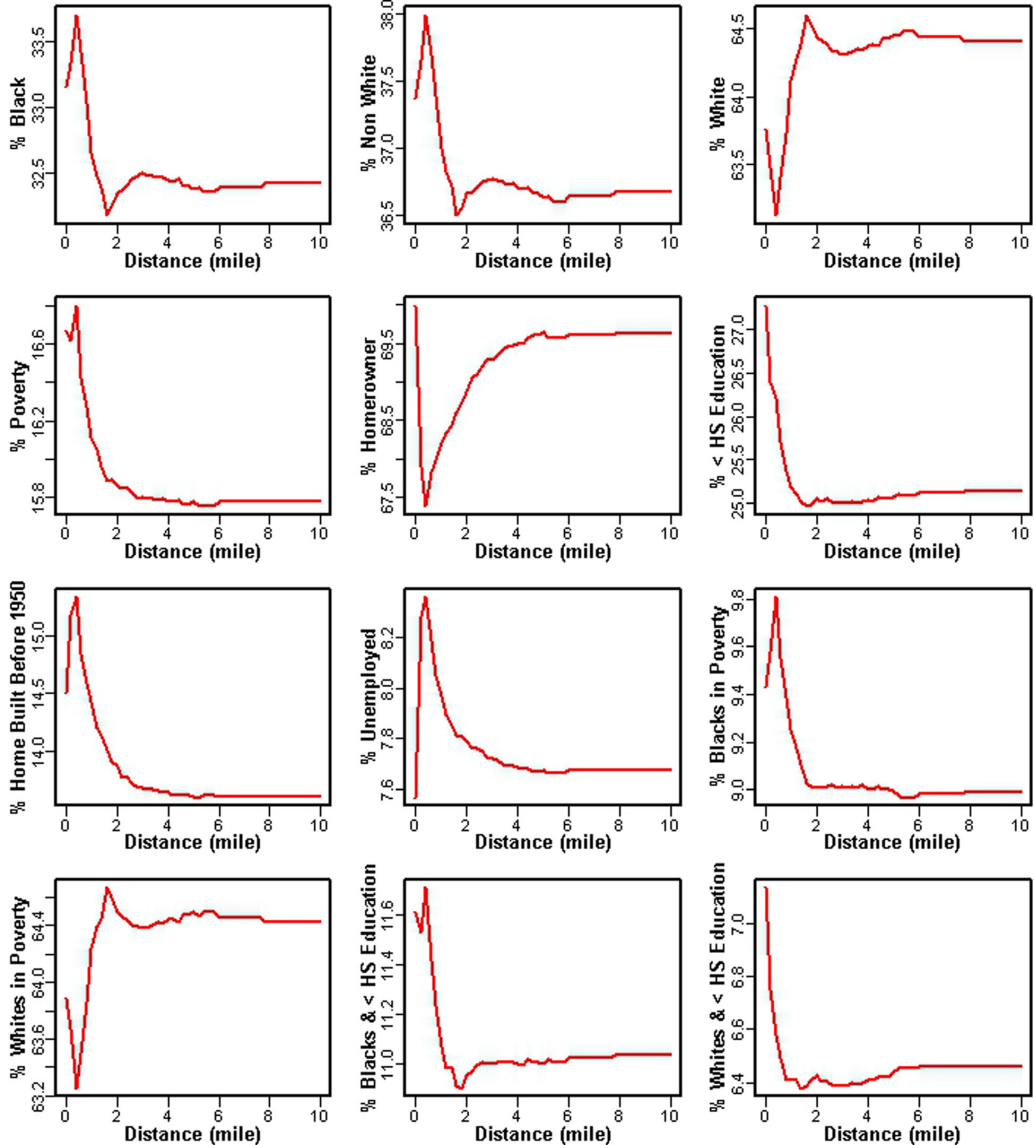
Association Between Sociodemographic Composition and Distance (miles) to the Nearest Superfund Site in South Carolina.

In all instances, as percent Black increased, the distance (both in km and miles) to the nearest Superfund site decreased. We observed similar results for the SES variables (% poverty, % unemployment, % with less than a high school education, % of Blacks in poverty, % of Whites in poverty, % Black with less than a high school education, and % White with less than a high school education). For example, the plots of distance to the nearest Superfund site for each sociodemographic factor (in miles) (Figure 3) illustrate that as the distance to the nearest Superfund site increased, the percentage of Blacks, Non-Whites, and SES measures (% poverty, % unemployment) decreased, while the % of Whites increased. We did not observe a clear pattern for the SES variable “% of people who own a home”. As a result, populations that are Black, Non-White, poor, or have less than a high school education may have a greater risk of being exposed to Superfund-related contamination.

Table 2 examines spatial disparities in the distribution of Superfund sites by comparing sociodemographic composition in host and non-host tracts. Overall, 54.8% of the population in SC lives within a Superfund host tract. Among the Black population (which accounted for 29.5% of the entire population), 55.9% lived within a Superfund host tract. There were similar findings for Non-Whites. In SC, 14.2% lived in poverty and 57.2% of this population lived within a Superfund host tract. Moreover, 59.2% of people with less than high school education (23.8% of the entire SC population), lived within host areas, and 54.5% of the unemployed lived in host areas even though the unemployed only account for 3.6% of the entire SC population. Among persons with less than high school education and living in Superfund host areas, 18.3% were Black, which is not statistically different from the corresponding percentage for Blacks living in non-host areas (18.8%, p = 0.1143). We did not find a statistically significant difference between percent blacks living in poverty in host areas (54.9%) and those in non-host areas (54.7%, p = 0.0625).

**Table 2:** Racial/ethnic and Socioeconomic Disparity for Superfund Host Versus Non-Host Census Tracts (US Census 2000)

**Demographics & Socioeconomic Variables**	Census Tracts*	Superfund Host Tracts **	Superfund Non-Host Tracts **
Population (%)	4,012,012	2,197,252 (54.8)	1,814,760 (45.2)
Black Population (%)	1,185,216 (29.5)	662,949 (55.9)	522,267 (44.1)
White Population (%)	2695560 (67.2)	1,464,784 (54.3)	1,230,776 (45.7)
Non-White Population (%)	1,316,452 (32.8)	732,468 (55.6)	583,984 (44.4)
Population Below Poverty Line (%)	567,783 (14.2)	324,523 (57.2)	243,260 (42.8)
Population Above Poverty Line (%)	3,444,229 (85.9)	1,872,729 (54.4)	1,571,500 (45.6)
Population with < HS Education (%)	953,243 (23.8)	564,396 (59.21)	388,847 (40.8)
Population with HS Education (%)	3,058,769 (76.2)	1,632,856 (53.4)	1,425,913 (46.6)
Unemployed Population (%)	146,043 (3.6)	79,559 (54.5)	66,484 (45.5)
Employed Population (%)	3,865,969 (96.4)	2,117,693 (54.8)	1,748,276 (45.2)

Note: * The percentages are with respect to the count 4,012,012. ** The percentages in each row are with respect to the count in the column “Census Tracts” in that row.

Chi-square analyses (Table 3) indicated that Blacks, Whites, Non-Whites or peopleof low SES were more likely to live in Superfund host areas than non-host areas. For instance, among people living in the host area, 30.2% were Black and 14.8% were living below poverty line, which was higher than the percentages (28.8%, and 13.4%, respectively) among people living in non-host areas. All results were significant at the multiple-testing adjusted significance level of 0.0036 (0.05/14.0 = 0.0036) with the exception of unemployment (p = 0.0230).

**Table 3:** Chi-square assessment of sociodemographic disparities in the distribution of superfund sites (Host Versus Non-Host Tracts)

**Variable**	**Host**	**Non-Host**	**Ratio of Host and Non-Host**	**p-value**
**Percent (%)**				
Black	30.2	28.8	1.05	<0.0001
Non-White	33.3	32.2	1.04	<0.0001
White	66.7	67.8	0.98	<0.0001
Poverty	14.8	13.4	1.10	<0.0001
Homeowners	73.1	71.4	1.02	<0.0001
< HS Education	25.7	21.4	1.20	<0.0001
Homes Built Before 1950	12.0	9.9	1.21	<0.0001
Unemployed	3.6	3.7	0.99	0.0230
Blacks in Poverty	26.9	25.5	1.06	<0.0001
Whites in Poverty	9.1	8.0	1.13	<0.0001
Blacks with < HS Education	7.7	7.5	1.03	<0.0001
Whites with < HS Education	7.7	6.3	1.23	<0.0001
**Mean (SD)**				
Per Capita Income	17739.5 (6500.5)	19371.7 (8157.7)	0.92	0.0012
Median HH Income	35085.1 (11403.4)	38446.5 (16526.8)	0.91	0.0005

Note: The percentages were calculated with respect to the host or non-host population sizes.

We created buffers for Superfund sites in SC census tracts based on the state’s total population of 4,012,012. In the 0.5mi buffer (figure not shown), the population was 30.3% Black and decreased to 29.6% and 29.5% for 1.0 and 5.0mi buffers, respectively. The Non-White population was approximately 33.6% for the 0.5mi buffer, which decreased to 32.9% and 32.8% for the 1.0 and 5.0mi buffers, respectively. In the 0.5km buffer, the population was 30.3% Black which decreased to 30.3% and 29.5% for 1.0 and 5.0km buffer, respectively. The Non-White population was roughly 33.6% for the 0.5km buffer, which decreased to 33.6% and 32.8% for 1.0 and 5.0km buffers. In contrast, the White population increased from roughly 66.0% in the 0.5km and 0.5mi buffers to 68.0% in the 5.0km and 5.0mi buffers. An analysis evaluating the distribution patterns of Superfund sites in relation to race/ethnicity and socioeconomic status across different buffers (in miles only) was performed. Based on the results, all ratios of host versus non-host were significantly different from 1, except for unemployment (Table 4), another indicator of burden disparity.

**Table 4:** Superfund host versus non-host ratios for race/ethnicity and socioeconomic variables in different buffers

**Variable**	**Host**	**0.5 Mile**	**1.0 Mile**	**5.0 Mile**
	Percent*	Ratio (p-value)**	Ratio (p-value)**	Ratio (p-value)**
Black	30.2	0.99 (<0.0001)	1.06 (<0.0001)	1.05 (<0.0001)
Non-White	33.3	0.97 (<0.0001)	1.04 (<0.0001)	1.04 (<0.0001)
White	66.7	1.01 (<0.0001)	0.98 (<0.0001)	0.98 (<0.0001)
Poverty	14.8	1.04 (<0.0001)	1.12 (<0.0001)	1.11 (<0.0001)
Owned Home	73.1	1.13 (<0.0001)	1.07 (<0.0001)	1.02 (<0.0001)
< HS Education	25.7	1.20 (<0.0001)	1.26 (<0.0001)	1.21 (<0.0001)
Homes Built Before 1950	12.0	0.99 (<0.0001)	1.12 (<0.0001)	1.21 (<0.0001)
Unemployed	3.6	0.90 (<0.0001)	0.96 (<0.0001)	0.99 (0.0807)
Blacks in Poverty	26.0	1.00 (0.2626)	1.04 (<0.0001)	1.05 (<0.0001)
Whites in Poverty	9.1	1.13 (<0.0001)	1.19 (<0.0001)	1.14 (<0.0001)
Blacks < HS Education	7.7	1.00 (0.8987)	1.06 (<0.0001)	1.04 (<0.0001)
Whites < HS Education	7.7	1.29 (<0.0001)	1.32 (<0.0001)	1.24 (<0.0001)
**Mean(SD)**				
Per Capita Income	17739.5 (6500.5)	0.96 (0.1657)	0.91 (0.0019)	0.91 (0.0007)
Median HH Income	35085.1 (11403.4)	0.95 (0.2063)	0.91 (0.0038)	0.91 (0.0003)

Note: *The percentages are with respect to the count 4,012,012. ** The p-values were from two sample proportion tests.

Linear regression models were applied to examine the association of distance to the nearest site (dependent variable) with all of the sociodemographic factors mentioned (independent variables). After adjusting for other sociodemographic factors noted earlier, % homeowners (p = 0.0008), % with less than a high school education (p = 0.0025), % who built their home before 1950 (p = 0.0271), % Black in poverty (p = 0.0165),% White with less than a high school education (p = 0.0108), and % Black with less than high school education (p < 0.0001) were statistically significant.

## Discussion

We found evidence of racial/ethnic, SES, and other disparities (homes built before 1950) in the spatial distribution of Superfund sites. Results from chi-square and linear regression analyses demonstrated a statistically significant difference in sociodemographic composition of populations living near Superfund sites, which were predominately Non-White, low-income, and less educated as well as those living in homes built before 1950. As percent Black and Non-White decreased, there was an inverse increase in buffer distance indicating that the percentage of these populations was higher at buffer distances closer to Superfund sites.

While per-capita (p < 0.0012) and median household income (p = 0.0005) were statistically significant and lower for populations who lived within Superfund host tracts, these differences were only found at 1.0mi, 5.0mi, and 5.0km buffer distances which may indicate that income may not be as important as other sociodemographic factors. There were also differences in the proportion of populations that host Superfund sites. While there were relatively small changes in percent Black and Non-White groups residing in each buffer zone, these populations were still located closer to a Superfund site than at distances farther away from a Superfund site.

While there was no statistical difference found between Superfund host and non-host tracts for Blacks with less than a high school education (p = 0.1143) and Blacks in poverty (p = 0.0625), there were still disparities found in the proportion of the population living within a host tract. Specifically, Black and Non-White populations, Black and White populations living in poverty, populations with a home built before 1950, and Black and White populations with less than a high school education were more likely to live in a Superfund host tract.

Moreover, backward selection results for % who own a home (p < 0.0008), % with less than a high school education (p = 0.0025), % White with less than a high school education (p = 0.0108) and % Black with less than high school education (p < 0.0001) were statistically significant which may indicate that these variables were the best predictors of whether a Superfund site would be present in a particular area.

The aforementioned results further support the claim that racial and SES disparities exist in the distribution of Superfund sites across the state at the census tract level. However, homeownership and populations who built their homes before 1950 are not necessarily determinants that are traditionally used in EJ research. As a result, further information may be needed to develop more comprehensive profiles for populations and communities that may be differentially burdened by Superfund sites and other environmental health hazards outside of the conventional variables used in EJ research.

Incorporating GIS and other spatial techniques into EJ research has become useful in demonstrating that spatial disparities exist in the distribution of environmental hazards and cumulative health risks [16-27]. However, burden disparities associated with the distribution of Superfund sites seems to be primarily a regulatory problem (i.e., procedural equity issue) because not only are environmental hazards disproportionately located in Non-White and low-income populations, Superfund sites in particular are less likely to undergo remediation if located within these communities [2]. A review by O’Neil provides evidence that the USEPA’s placement of hazardous sites on the NPL has been slow due in part to poor implementation of the Executive Order 12898 at the regional level [2]. Furthermore, the use of GIS to spatially describe disparities is limited by our knowledge of the location of Superfund sites because not all hazardous waste sites have been placed on the NPL. The lack of placement of these sites on the NPL in EJ communities can limit the availability of resources needed for revitalization efforts.

The findings from this study are unique because no previous studies have used spatial methods to assess the distribution of Superfund sites across the state of SC. Similar studies conducted by Wilson et al. [26,27] on the distribution of toxic release inventory (TRI) facilities and leaking underground storage tanks (LUSTs) in Charleston, SC found that there was a higher prevalence of Black and Non-White populations in census tracts and blocks that host LUSTs and TRI facilities versus those not hosting these environmental hazards. The cumulative impact of these sites on EJ communities throughout the state may have serious health and environmental implications.

In EJ communities, there are underlying vulnerabilities experienced at the individual or population levels that may modify the effect of exposure to contaminants released from Superfund sites and contribute to higher health risks for local residents. While these communities may be exposed to toxic releases from Superfund sites, they may also experience social stressors due to limited access to high quality health-promoting infrastructure, overcrowding, and poverty [28-34]. These pathogenic conditions in combination with exposure to Superfund-related emissions may contribute to environmental health disparities [18,28-31,34].

## Conclusion

Despite the paucity of literature available on environmental disparities related to the distribution of Superfund sites in SC, this study has shown that there are burden disparities in the location of these sites for Non-White and low-income populations at the block and census tract levels. While there were some limitations in the methodology, the study found significant differences in the proportion of populations living within a Superfund host and non-host census tract for Black and Non-White populations, populations below povertyline, populations with less than a high school education, populations with a home built before 1950, unemployed persons, Black and White populations in poverty, as well as Black and White populations with less than a high school education.

This information may be useful to community-based organizations (CBOs) seeking to obtain information on the spatial distribution of Superfund sites and assistance from federal agencies such as the USEPA and the Agency for Toxic Substances and Disease Registry (ATSDR) to study the negative health impacts of these sites as part of a comprehensive community revitalization program such as the program implemented by the ReGenesis project in Spartanburg, SC [35,36]. In addition, we suggest that CBOs that represent residents who live near Superfund and NPL sites work with state agencies such as the SC Department of Health and Environmental Control (SCDHEC) to use the results of this study to prioritize hazardous sites in vulnerable communities and leverage state resources to remediate those sites. CBOs such as the Lowcountry Alliance for Model Communities (LAMC) and ReGenesis can work with the SCDHEC and impacted communities using the USEPA’s EJ collaborative problem-solving model (CPS) to remediate sites of concern in the state of SC.

## Abbreviations

ATSDR: Agency for toxic substances and disease registry; CERCLIS: Comprehensive environmental response, compensation, and liability information; GIS: Geographic information systems; LUSTs: Leaking underground storage tanks; NPL: National priorities list; SC: South Carolina; SCDHEC: South Carolina department of health and environmental control; SES: Socioeconomic status; TRI: Toxic release inventory; USEPA: United States environmental protection agency; VOCs: Volatile organic compounds; ZCTAs: ZIP code tabulation areas.

## Competing interests

There are no financial or non-financial competing interests to disclose.

## Authors’ contributions

KB-N drafted the manuscript. HZ helped with design and performed the statistical analysis. CJ assisted with statistical analysis and mapping. AS assisted with statistical analysis. LD assisted with drafting the introduction. LR assisted with revising the manuscript. ED assisted with revising the manuscript. SW designed the study and assisted with drafting the manuscript. All authors read and approved the final manuscript.
